# Clinical manifestations and associated factors in acquired hypoaldosteronism in endocrinological practice

**DOI:** 10.3389/fendo.2022.990148

**Published:** 2022-10-11

**Authors:** Jorge Gabriel Ruiz-Sánchez, Alfonso Luis Calle-Pascual, Miguel Ángel Rubio-Herrera, María Paz De Miguel Novoa, Emilia Gómez-Hoyos, Isabelle Runkle

**Affiliations:** ^1^ Servicio de Endocrinología y Nutrición, Instituto de Investigación Sanitaria Fundación Jiménez Díaz (IIS-FJD, UAM), Hospital Universitario Fundación Jiménez Díaz, Madrid, Spain; ^2^ Facultad de Medicina, Universidad Complutense de Madrid, Madrid, Spain; ^3^ Servicio de Endocrinología y Nutrición, Instituto de Investigación Sanitaria del Hospital Clínico San Carlos (IdISSC), Hospital Clínico San Carlos, Madrid, Spain; ^4^ Centro de Investigación Biomédica en Red de Diabetes y Enfermedades Metabólicas Asociadas (CIBERDEM), Madrid, Spain; ^5^ Servicio de Endocrinología y Nutrición, Hospital Clínico Universitario de Valladolid, Valladolid, Spain

**Keywords:** hypoaldosteronism, hypovolemic hyponatremia, hyperkalemia, metabolic acidosis, isolated hypoaldosteronism

## Abstract

**Introduction:**

Hypoaldosteronism can be congenital or acquired, isolated or part of primary adrenal insufficiency, and caused by an aldosterone deficit, resistance, or a combination of both. Reduced mineralocorticoid action can induce a decrease in urine K+ and H+ excretion and an increase in urine Na+ excretion, leading to hyperkalemia, and/or hyponatremia, often combined with metabolic acidosis. We aimed to characterize the clinical manifestations of hypoaldosteronism, and their associated factors.

**Methods:**

Retrospective analysis of 112 episodes of hypoaldosteronism diagnosed in 86 adult patients from 2012-2019 by the Endocrinology and Nutrition Department of a tertiary hospital. The frequency of hyperkalemia, hypovolemic hyponatremia (HH) and metabolic acidosis (MA), and their associated factors were evaluated.

**Results:**

Patients had a median age of 77 [65 – 84], 55.4% were male. 94.6% cases showed hyperkalemia, 54.5% HH, and 60.3% MA. The mean serum K+ of all cases was 5.4 ± 0.5 mmol/L, Na+: 132.1 ± 6.3 mmol/L, HCO3: 22.6 ± 3.3 mmol/L. Hypoaldosteronism was isolated in the majority of cases: only 6/112 (5%) had primary adrenal insufficiency. Hypovolemia was associated with hyponatremia and a more florid clinical presentation. HH was associated with a combined presence of aldosterone-lowering and mineralocorticoid resistance factors. MA was associated with the presence of mineralocorticoid resistance factors.

**Conclusions:**

Hypoaldosteronism in adult endocrinological clinical practice is primarily isolated, and acquired. It predisposes not only to the development of hyperkalemia and MA, but also to that of HH. Hypoaldosteronism must be considered in the differential diagnosis of HH with urinary sodium wasting.

## Introduction

Hypoaldosteronism is a clinical condition caused by a deficit in the action of the principal human mineralocorticoid, the adrenal hormone aldosterone (1). Hypoaldosteronism can be congenital or acquired, isolated or part of primary adrenal insufficiency (PAI). It can be induced by an aldosterone deficit, resistance, or a combination of both elements (2, 3). When aldosterone resistance is present, the term pseudo-hypoaldosteronism has been used, particularly when congenital.

Mineralocorticoids act on the nephron primarily through stimulus of the mineralocorticoid receptor (MR) in principal cells of the distal-convoluted tubule and the collecting duct, as well as the intercalated alpha cells of the latter. Mineralocorticoid principal-cell action induces synthesis of amiloride-sensitive epithelial Na+ channels, and their insertion in the lumen of the distal tubule and collecting duct, as well as the activity of the capillary-side Na+/K+ ATPase pump. Sodium is absorbed from the tubular lumen, and passes into the blood, exchanged for blood potassium, which is then secreted into the lumen with H+. The intercalated cells, however, are more important for acid-base equilibrium, as mineralocorticoids promote an increase in urinary H+ excretion, and in bicarbonate secretion into the bloodstream. Although normally aldosterone is the primary ligand of the principal-cell MR, cortisol acquires importance upon reaching levels in the upper-range-of-normal (4).

The direct result of insufficient mineralocorticoid action on these principal cells is an inadequate reduction in urinary potassium excretion. In fact, the latter is determined primarily by mineralocorticoid action, absent oliguric renal insufficiency, if sodium delivery to the distal nephron is sufficient (1). This potassium excretion deficit predisposes to the development of hyperkalemia, the most salient characteristic of hypoaldosteronism (5), albeit not a constant (1). Thus, reduced potassium excretion is the defining characteristic of hypoaldosteronism, rather than hyperkalemia itself. The inability to reabsorb intraluminal sodium in the distal nephron also induces varying degrees of renal sodium loss, and can result in hypovolemia and hyponatremia, as occurs in salt-wasting. Furthermore, decreased principal cell H+ excretion together with reduced alpha-intercalated-cell function may cause metabolic acidosis, such as type 4 renal tubular acidosis (4RTA).

Since hypoaldosteronism was first described in 1957 (6), the case-series published have included limited numbers of patients. DeFronzo´s 1980 recompilation, the largest to date, included 81 cases from 35 different studies (5). Currently, the incidence and prevalence of hypoaldosteronism are unknown. However, Hass et al. detected 4RTA in 4% of hospitalized patients, as fully 48% of detected cases of severe hyperkalemia (7). Furthermore, a review of Wilczynski et al. found that hypoaldosteronism was the cause of between 10% to close to 80% of the published case-series of hyperkalemia (8). Therefore, given hyperkalemia is common in clinical practice, hypoaldosteronism may be a frequent disorder, albeit underdiagnosed.

In spite of progress in the understanding of mineralocorticoid physiology (9–13), there is a dearth of studies identifying hypoaldosteronism’s clinical manifestations, and their relative frequency (8, 14, 15). In fact, classically isolated hypoaldosteronism has been primarily related with hyperkalemia or 4RTA, but not with hyponatremia, despite the fact that the increase in urinary sodium loss characteristic of this condition per se predisposes to hyponatremia and hypovolemia. Thus, hypoaldosteronism-induced hyponatremia has not been a focus of research, its relative frequency and the factors associated with its presence yet to be described.

Acquaintance with the clinical spectrum and risk factors associated with hypoaldosteronism permits the clinician to promptly detect and in some cases prevent the development of this relatively frequent, yet underdiagnosed condition. Furthermore, undetected hypoaldosteronism, in our experience, can lead to the progressive development of severe hyperkalemia and/or hyponatremia. Yet the treatment of isolated hypoaldosteronism is relatively straight-forward: precipitating medication must be discontinued, and specific therapy with fluid and salt commenced (with oral salty broths, intravenous isotonic or hypertonic saline solution), and with mineralocorticoids (primarily fludrocortisone) associated when needed. With an expeditious diagnosis, therapy can then be rapidly initiated, and the electrolyte and acid-base alterations corrected in a timely manner. Yet precipitating medication is not always recognized as such, in spite of the fact that the use of drugs that interfere with components of the renin-angiotensin-aldosterone system (RAAS) is relatively commonplace. These drugs include, but are not limited to, first-line hypertension medication. However, the frequency of the presence of RAAS- modifying pharmacological agents in patients developing hypoaldosteronism has still to be described, being currently unknown.

The need for an increased knowledge base of the characteristics of hypoaldosteronism that would permit its prompt diagnosis and therapy has led to the analysis presented in the current study. We sought to describe the clinical spectrum of hypoaldosteronism, as well as risk factors for their apparition, in a series of patients diagnosed with this disorder by the Endocrinology and Nutrition Department (END) of a tertiary hospital.

## Subjects and methods

### Study design

We retrospectively studied all patients ≥ 18 years of age diagnosed with hypoaldosteronism from January 1^st^, 2012, through August 19^th^, 2019, registered by the END of the Hospital Clínico San Carlos of Madrid (HCSC). All patients were assessed by END physicians on hospital wards or at an outpatient clinic, following detection of hyponatremia and/or hyperkalemia.

The study complied with accepted standards of good clinical practice according to the Helsinki Declaration and was approved by the Ethical Committee of the HCSC (Cod. 20/714-E_BS, December 14^th^-2020). Written informed consent was waived.

### Subjects

**Figure 1:**
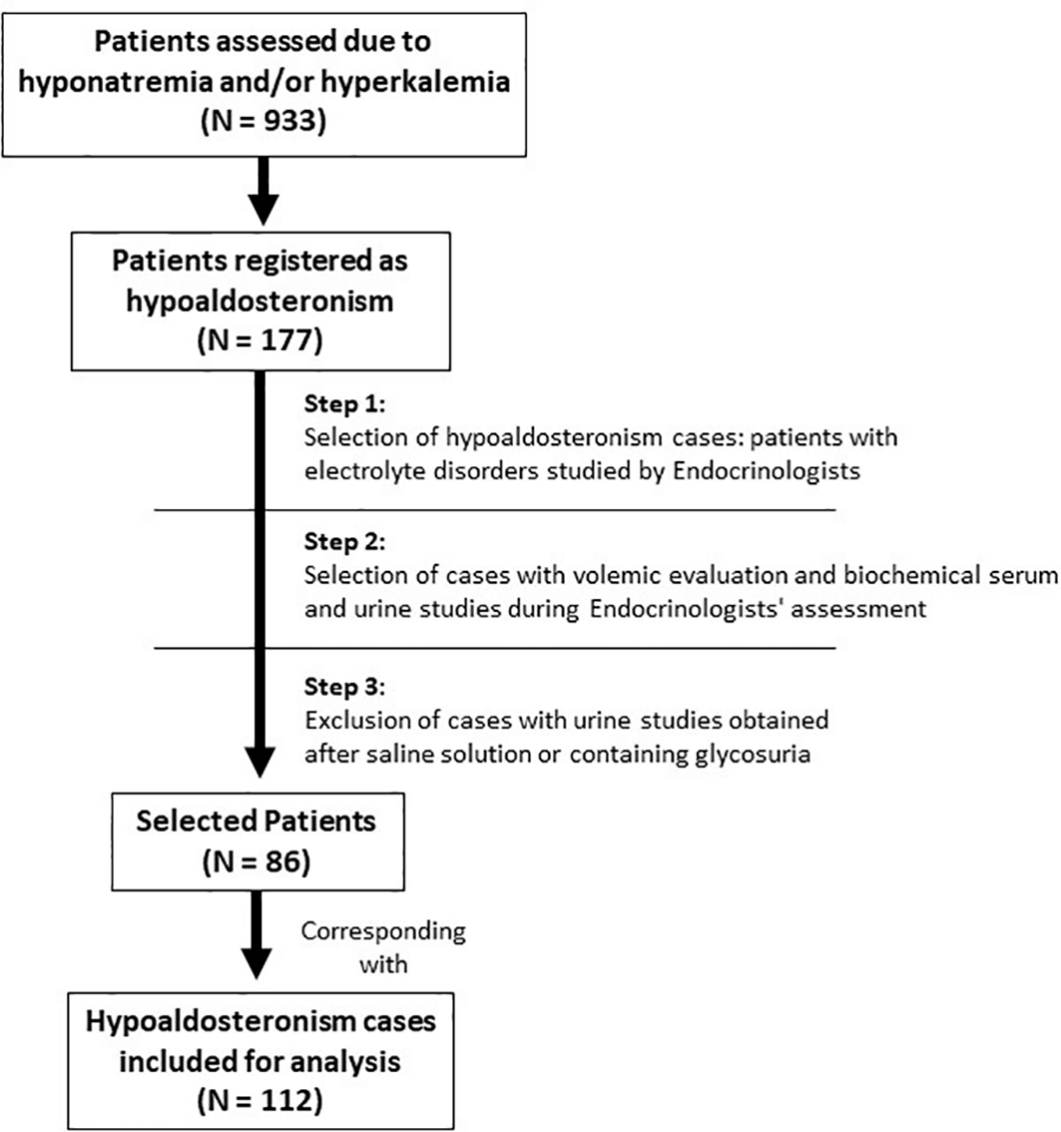
Algorithm of selection.

Of 933 patients evaluated (893 for hyponatremia with or without hyperkalemia, and 40 solely for the latter), a total of 177 patients were diagnosed with hypoaldosteronism. Their clinical records were reviewed by our research team, and the diagnosis of hypoaldosteronism ratified or discarded in compliance with the criteria described in **Table 1**. Patients with transitory hypoaldosteronism following adrenal surgery for primary hyperaldosteronism or with hypoaldosteronism secondary to excessive doses of eplerenone or spironolactone in the therapy of primary hyperaldosteronism were excluded, as were patients with hypervolemia (heart failure, cirrhosis, third space) or oliguric renal failure. The process of patient selection for inclusion is displayed in **Figure 1**. When the same patient was assessed for hyponatremia and/or hyperkalemia more than once, with a minimum of 6 months elapsing between evaluations, and confirmation of interim eunatremia and eukalemia, each episode was considered to be a different case. Thus, of the 177 patients, 86 were finally included, corresponding with 112 cases (episodes) for analysis.

**Table 1:** Criteria used for diagnosis of hypoaldosteronism.

**A**. Non-fictitious persistent hyperkalemia: -At least 2 laboratory tests with hyperkalemia performed on different days -In addition to: —Absence of an external potassium load —Glomerular filtration rate > 30 ml/min/1.73 m^2^
**B.** Hypovolemic hyponatremia with elevated urine sodium (≥ 30 mmol/L) persisting after diuretic withdrawal (at least five half-lives having elapsed), and having ruled out: -Bicarbonate administration -Cerebral salt-wasting -A proximal tubular disorder
**C.** Absence of hypokalemia
**D.** An adequate therapeutic response: -Improvement of the electrolyte disorder(s) with mineralocorticoid replacement therapy and/or volume and salt repletion with isotonic saline, hypertonic saline, or increased water and salt intake.
Diagnosis required the presence of **A** and/or **B**, together with **C** and **D**.

### Data collection

For each episode, clinical data was collected from the moment in which a complete END assessment was performed and diagnosis of hypoaldosteronism established. These data referred to the medical history, concomitant treatment, volemic status, blood and urine biochemistry. Biochemical and volemic data from the day of the measurement of serum aldosterone (ALD) was recorded when available. Zenith values of serum potassium (SK) and nadir values of serum sodium (SNa) during the evaluated episode were also registered. Collected clinical and biochemical variables are shown in **Supplement**.

Variables from medical history were as follows: subjective self-reported low-sodium diet, a prior diagnosis of PAI, hypertension, diabetes mellitus (DM), chronic kidney disease [(CKD), if glomerular filtrate rate ≤ 60 ml/min/1.73 m^2^, [CKD-EPI equation (16)], obstructive uropathy, renal transplant, urinary tract infection, malnutrition, a history of alcoholism, and a prior history of dilutional hyponatremia [(PDH), defined as a hyponatremia event accompanied by a descent in serum creatinine (SC) as compared to a prior eunatremic SC].

Variables from concomitant treatment were: the use of heparin, trimethoprim, cyclosporine, tacrolimus, no-steroid anti-inflammatories (NSAID), beta blockers (βB), aliskiren, angiotensin-converting enzyme inhibitors (ACEI) or angiotensin 2 receptor blockers (ARB), mineralocorticoid receptor blockers (MRB), loop diuretics, thiazides, thiazide-amiloride diuretics, short-term glucocorticoid-therapy (when prednisone, prednisolone or dexamethasone were initiated during the episode or the preceeding 6 weeks), and chronic glucocorticoid-therapy (if it was initiated ≥ 6 weeks before the episode).

### Clinical definitions

Diagnosis of hypoaldosteronism was based on the criteria given in **Table 1**. Hypoaldosteronism was classified as isolated if PAI was ruled out. If PAI was not previously known, it was diagnosed when 8 AM cortisol was < 15 µg/dL together with an ACTH above the upper laboratory range (> 40 pg/ml), or cortisol < 18 µg/dL after 250 µg 1-24 ACTH stimulus test in absence of glucocorticoid-therapy. Hypoaldosteronism-associated metabolic acidosis (MA) was diagnosed when blood bicarbonate (HCO3) was < 23 mmol/L accompanied by a whole blood venous or arterial pH <7.36, or pH ≥7.36 with PCO2 <35 mmHg, in both cases in the presence of a normal anion gap (17). ALD levels measured coinciding with hyperkalemia were used for the differential diagnosis of hypoaldosteronism based on its causal mechanism as follows: secondary to an aldosterone deficit (AD) if ALD was < 90 pg/mL, an aldosterone/mineralocorticoid resistance (MRes) if ALD was > 200 pg/mL, or a combination of both (CB) if ALD was between 90-200 pg/mL.

Hyperkalemia was defined as a SK ≥ 5mmo/L, hypokalemia when SK was ≤ 3.5 mmol/L, and hyponatremia as a SNa ≤ 135 mmol/L. SNa was corrected for glycemia (18) when the latter was ≥ 140 mg/dL. Hyponatremia was classified as hypovolemic (HH) if the maximum height of the internal jugular pulse (HIJP) was below the sternal angle with the patient reclined at 0-30°, in addition to at least two of the following data suggesting hypoperfusion: thirst, orthostatic symptoms/signs, blood pressure ≤ 90/60 mmHg, heart rate ≥ 90 bpm, decreased eye tone on palpation, distal venous filling of the upper limbs below the diaphragmatic line in a sitting position, a rise in serum creatinine (SC) accompanying the descent in SNa (19, 20). When the HIJP was not measured, hypovolemia was determined by the presence of at least three of the other signs/symptoms described above. Patients without symptoms/signs of hypovolemia and with a HIJP at 1-3 cm above the sternal angle were classified as euvolemic.

Comorbidities and drugs/therapy were classified as “non-modifiable” or “modifiable factors”, and as “aldosterone-lowering factors” (ALowF), “mineralocorticoid-resistance factors” (ResF), or any combination of both (CombF) according to their interaction with mineralocorticoid homeostasis. Drugs were grouped according to their ability to interfere with the RAAS (**Details in Supplement**).

### Analysis

Categorical variables are described as frequencies and percentages, parametric variables as mean and standard deviation (±), with non-parametric variables described as the median and interquartile range (in brackets). Ranges are given when relevant. The parametric tendency of quantitative variables was analyzed with the Kolmogorov-Smirnov or Shapiro-Wilk tests. Comparative analyses of the qualitative variables were performed using Chi-squared, Fisher´s, or likelihood ratio tests. Mann-Whitney U or Kruskal-Wallis tests were used in case of non-parametric, and T-student or ANOVA tests in case of parametric variables.

Correlation coefficients (r) of quantitative variables were calculated with Pearson`s or Spearman`s method according to parametric tendency. Univariate and multivariate logistic regression analyses (MLRA) were performed, and Odds ratios (OR) calculated. MLRA were executed with Wald´s method of steps forward. MLRA models included age, sex, and those variables with a p value <0.1 in the univariate analysis. OR were calculated with ninety-five percent confidence intervals (95%CI). A two-tailed p value <0.05 was considered to be statistically significant. SPSS version 25 (IBM Corp., N.Y., USA) was used for the statistical analysis.

ALD was measure by radioimmunoassay, serum, and urinary electrolytes with AU5800^®^ Analyzer (Beckman Coulter) by indirect potentiometry (inter-assay variability coefficient <0.01) or direct whole blood gas analysis. POsm and UOsm were measured by A2O^®^ osmometer (Advanced Instruments, INC.) by freezing point.

## Results

Of the 112 cases of hypoaldosteronism, 62 (55.4%) were male. The median age was 77 years [65 – 84]. Thirty-seven (33%) cases were evaluated on hospital wards, the rest at outpatient clinic. All cases were treated with increased oral fluid and salt intake, or isotonic or hypertonic saline when hypovolemia was present. Twelve cases (10.7%) also received fludrocortisone or hydrocortisone therapy.

### Clinical manifestations

At diagnosis, 106/112 (94.6%) cases had hyperkalemia, 71/112 (63.4%) hypovolemia, 80/112 (71.4%) hyponatremia, and MA was observed in 35 of the 58 cases (60.3%) with acid-base status available. Hyponatremia coincided with hyperkalemia in the majority of cases: 74/112 (66.1%). The hyponatremia was primarily hypovolemic: 61/80 (76%), and in 61/112 (54.5%) of total cases. HH coincided with hyperkalemia in 55/61 (90%). Hyperkalemia was undetected throughout the episode in 6/61 (9.8%) HH cases and in 6/112 (5.4%) of total cases. The description of age, sex, presence of MA, comorbidities, and concomitant treatment at diagnosis in all cases, as well as their description according to the presence of hyperkalemia or HH at the time of diagnosis is shown in **Table 2**.

**Table 2:** Medical history and concomitant treatment at diagnosis of the cases according to the presence of hyperkalemia and hypovolemic hyponatremia.

	TOTAL	Hyperkalemia		*p^1^*	Hypovolemic Hyponatremia		*p^2^*
	N=112	No (N=6)	Yes (N=106)		No (N=51)	Yes (N=61)	
Age, years ≥ 65 y, n (%)	77 [65-84] 87 (77.7)	86 [76-90] 6 (100)	76 [65-84] 81 (76.4)	*0.177* *0.335*	75 [63-84] 36 (70.6)	78 [70-84] 51 (83.6)	*0.241* *0.099*
Male, n (%)	62 (55.4)	1 (16.7)	61 (57.5)	*0.087*	29 (56.9)	33 (54.1)	*0.769*
Metabolic acidosis, n (%)	35/58 (60.3)	1/1 (100)	34/57 (59.6)	*1*	15/29 (51.7)	20/29 (69)	*0.180*
COMORBIDITIES							
Hypertension, n (%)	85 (75.9)	6 (100)	79 (74.5)	*0.333*	36 (70.6)	49 (80.3)	*0.230*
Low sodium diet, n (%)	53 (47.3)	4 (66.7)	49 (46.2)	*0.42*	22 (43.1)	31 (50.8)	*0.417*
DM, n (%) >10 y	59 (52.7) 35 (31.3)	2 (33.3) 0	57 (53.8) 35 (33)	*0.329* *0.174*	31 (60.8) 21 (41.2)	28 (45.9) 14 (23)	*0.116* *0.038*
CKD, n (%)	39 (34.8)	1 (16.7)	38 (35.8)	*0.663*	19 (37.3)	20 (32.8)	*0.621*
Obstructive uropathy, n (%)	29 (25.9)	0	29 (27.4)	*0.336*	10 (19.6)	19 (31.1)	*0.165*
Urine infection, n (%)	10 (8.9)	0	10 (9.4)	*1*	3 (5.9)	7 (11.5)	*0.342*
Renal transplant, n (%)	5 (4.5)	0	5 (4.7)	*1*	2 (3.9)	3 (4.9)	*1*
Prior Dilutional hyponatremia, n (%)	67 (59.8)	3 (50)	64 (60.4)	*0.614*	25 (49)	42 (68.9)	*0.033*
Malnutrition, n (%)	38 (33.9)	3 (59)	35 (33)	*0.406*	8 (15.7)	30 (49.2)	*<0.001*
Chronic alcoholism, n (%)	9 (8)	0	9 (8.5)	*1*	5 (9.8)	4 (6.6)	*0.729*
Primary adrenal insufficiency, n (%)	7 (7.8)	0	7 (8.2)	*1*	3 (7.7)	4 (7.8)	*1*
CONCOMITANT PHARMACOLOGICAL TREATMENT							
ACEI/ARB, n (%)	57 (50.9)	5 (83.3)	52 (49.1)	*0.206*	25 (49)	32 (52.5)	*0.717*
MRB, n (%)	13 (11.6)	0	13 (12.3)	*1*	1 (2)	12 (19.7)	*0.006*
Short-term GC, n (%)	21 (18.8)	1 (16.7)	20 (18.9)	*1*	5 (9.8)	16 (26.2)	*0.027*
Chronic GC, n (%)	16 (14.3%)	1 (16.7)	15 (14.2)	*1*	8 (15.7)	8 (13.1)	*0.699*
Diuretics, n (%)	34 (30.4)	3 (50)	31 (29.2)	*0.365*	10 (19.6)	24 (39.6)	*0.024*
Loop diuretic, n (%)	24 (21.4)	2 (33.3)	22 (20.8)	*0.607*	8 (15.7)	16 (26.2)	*0.176*
Thiazide Thiazide + amiloride, n (%)	11 (9.8) 2 (1.8)	1 (16.7) 0	10 (9.4) 2 (1.9)	*0.470* *1*	2 (3.9) 0	9 (14.8) 2 (3.3)	*0.064* *0.5*
Heparin, n (%)	28 (25)	1 (16.7)	27 (25.5)	*1*	8 (15.7)	20 (32.8)	*0.037*
Trimethoprim, n (%)	14 (12.5)	0	14 (13.2)	*1*	4 (7.8)	10 (6.4)	*0.252*
Tacrolimus, n (%)	5 (4.5)	0	5 (4.7)	*1*	2 (1.8)	3 (2.7)	*1*
Cyclosporine, n (%)	1 (0.9)	0	1 (0.9)	*1*	0	1 (1.6)	*1*
NSAID, n (%)	9 (8)	0	9 (8.5)	*1*	4 (7.8)	5 (8.2)	*1*
β-blockers, n (%)	20 (17.9)	1 (16.7)	19 (17.9)	*1*	9 (17.6)	11 (18)	*0.958*

HH, hypovolemic hyponatremia; DM, diabetes mellitus; CKD, chronic kidney disease; ACEI, angiotensin-converting enzyme inhibitors; ARB, angiotensin-2 receptor blockers; MRB, mineralocorticoid receptor blockers; short-term GC, less than 6 weeks of glucocorticoid therapy; chronic GC, ≥ 6 weeks of glucocorticoid therapy; NSAID, non-steroid anti-inflammatory drugs.

1. Eukalemia versus hyperkalemia.

2. Hypovolemic hyponatremia vs non-hypovolemic hyponatremia.

At diagnosis, mean SK of all cases was 5.4 ± 0.5 mmol/L, and mean SNa 132.1 ± 6.3 mmol/L. The mean HCO3 was 22.6 ± 3.3 mmol/L. Upon reviewing the entire episode assessed by END, we found a mean zenith SK of 5.8 ± 0.5 mmol/L (range: 4.2-7.3) and a mean nadir SNa of 127.5 ± 7.5 (range: 108-143). Urine parameters of all cases were indicative of low/inadequate K+ excretion regardless of the presence or absence of HH (**Table 3**). Furthermore, we found significant correlations between these urine markers of K+ excretion as follows: the trans-tubular potassium gradient (TTKG) with urinary K+/creatinine ratio (r=0.327; p=0.019), TTKG with urinary Na+/K+ ratio (r=-0.69; p<0.001), TTKG with fractional potassium excretion (FKE) (r=0.476; p<0.001), and urinary K+/creatinine ratio with FKE (r=0.643; p<0.001). Biochemical blood and urine parameters and their comparison according to the presence or absence of HH are described in **Table 3**.

**Table 3:** Biochemical parameters at diagnosis in all cases compared according to the presence or absence of hypovolemic hyponatremia.

	Total (N = 112)	Hypovolemic hyponatremia(N = 61)	Absence of Hypovolemic hyponatremia (N=51)	*p*
Serum Na, mmol/L	132 ± 6	129 ± 5	136 ± 5	<0.001*
Serum K, mmol/L	5.4 ± 0.5	5.4 ± 0.6	5.4 ± 0.4	*0.945*
Serum Creatinine, mg/dL	1.2 ± 0.5	1.2 ± 0.5	1.1 ± 0.5	*0.386*
GFR, ml/min/1.73m^2^	115 ± 40	111 ± 41	120 ± 40	*0.218*
HCO3, mmol/L	22.6 ± 3.3	21.9 ± 3.3	23 ± 3.1	*0.123*
Cortisol, µg/dL	16.2 ± 5.2	15.9 ± 6	16.5 ± 3.9	*0.565*
Serum K/urine K ratio	0.19 [0.15-0.25]	0.2 [0.15-0.24]	0.19 [0.14-0.27]	*0.603*
Fractional excretion of K	13.4 ± 7.7	14.4 ± 6.8	12.5 ± 8.3	*0.338*
Urine K/creatinine ratio, mmol/L/mg/dL	60.9 ± 28	66.3 ± 30.7	56.3 ± 25	*0.158*
Urine Na, mmol/L	79 ± 38	71 ± 38	89 ± 36	0.014*
Urine K, mmol/L	30 ± 12	30 ± 11	30 ± 13	*0.923*
Urine Na/k ratio	2.9 ± 1.5	2.6 ± 1.6	3.28 ± 1.38	0.027*
Uosm, mOsm/kg	419 ± 140	401 ± 138	443 ± 142	*0.127*
TTKG	3.7 [3.1-4.6]	3.7 [3.4-4.7]	3.7 [2.7-4.5]	*0.907*

Na, sodium; K, potassium, HCO3: bicarbonate; Uosm, urine osmolality; TTKG, trans-tubular potassium gradient.

*p<0.005.

### Impact of volemic status on blood potassium, sodium, and bicarbonate

Seventy one of the 112 cases (63.4%) were hypovolemic, regardless of SNa levels. Similar values of SK were found in the hypovolemic when compared with euvolemic: 5.4 ± 0.5 vs. 5.3 ± 0.4 mmol/L (p=0.477). Hypovolemic cases had lower mean values of SNa than those euvolemic: 130.3 ± 6 vs. 135.2 ± 5 mmol/L (p<0.001). Mean HCO3 values tended to be lower in hypovolemic than euvolemic: 22 ± 0.5 vs. 23.7 ± 3 mmol/L (p=0.058).

Cases with SK ≥ 6 mmol/L, SNa ≤ 125 mmol/L, and HCO3 ≤ 20 mmol/L were more frequently hypovolemic than euvolemic: 90% vs. 10% (p=0.094), 88.9% vs. 11.1% (p=0.029), and 33.3% vs. 9.5% (p=0.058), respectively. Hypovolemia was associated with the presence of hyponatremia (p<0.001), with only 10/71 (14.1%) hypovolemics not showing concomitant hyponatremia. Although a higher rate of MA was found in hypovolemics than in euvolemics (66.7% vs. 47.6%), the difference was not statistically significant (p=0.157). However, hypovolemia was associated with the combined presence of hyperkalemia, HH and MA (p<0.001), and all 19 cases where these 3 manifestations concurred were hypovolemic.

### Clinical characteristics of the non-hyperkalemic cases

An individual description of comorbidities, concomitant treatment, and biochemical data at diagnosis of the 6 cases in which hyperkalemia was not detected at any time during the hypoaldosteronism episode is presented in **Table 4**. None were receiving short-term glucocorticoid-therapy, nor medication associated with mineralocorticoid resistance.

**Table 4:** Description of clinical data at diagnosis of hypoaldosteronism cases without hyperkalemia.

	Sex/Age y	Comorbidities	Concomitant treatment	SK^1^	SNa^1^	SC^2^	GFR^3^	HCO3^1^	SK/UK ratio	FEK	UK/UC ratio	UNa^1^	UK^1^	UNa/uK ratio	Uosm^4^	TTKG	Cortisol^5^	PAC^6^
Case 1	F/68	HTA, PDH	ACEI/ARB	4.2	130	0.77	150	NM	0.14	9.5	51.9	81	31	2.61	490	4.2	14.8	88
Case 2	F/83	HTA, PDH, MN	Hep, ACEI/ARB, TD	3.6	118	0.46	186	NM	0.11	NM	NM	60	32	1.88	527	4.4	29	7
Case 3	F/89	DM, HTA, LSI	ACEI/ARB, LD	4.4	134	1.17	103	NM	0.23	11.2	42.1	60	19	3.16	285	4.3	15.9	NM
Case 4	M/78	HTA, PDH, LSI	ACEI/ARB	4.6	126	1.36	86	NM	0.18	NM	NM	68	25	2.72	294	5	16.3	218
Case 5	F/90	DM, HTA, MN, LSI	CG, βB, ACEI/ARB, LD	4.2	124	0.97	129	NM	0.10	NM	NM	36	41	0.88	514	5.3	NM	NM
Case 6	F/90	HTA, CKD, RT, MN, LSI	ACEI/ARB	4.3	127	1.17	75	22.3	0.19	4.8	17.6	36	23	1.57	312	4.6	21	83

F, female; M, male; HTA, hypertension; DM, diabetes mellitus; CKD, chronic kidney disease; RT, renal transplantation; PDH, prior dilutional hyponatremia; MN, malnutrition; low-salt intake; ACEI/ARB, angiotensin-converting enzyme inhibitors or angiotensin-2 receptor blockers; Hep, heparin; TD, thiazide; LD, loop diuretics; CG, chronic glucocorticoid therapy ≥ 6 weeks; βB, beta-blockers; NM, no measured. SK, serum potassium mmol/L; SNa, serum sodium; SC, serum creatinine; GFR, glomerular filtrate rate; HCO3, blood bicarbonate; FEK, fractional excretion of K; UK, urine potassium; UC, urine creatinine; UNa, urinary sodium; Uosm, urine osmolality; TTKG, transtubular potassium gradient.

^1^mmol/L, ^2^mg/dL, ^3^ml/min/1.73m^2^, ^4^mOsm/kg, ^5^µg/dL, ^6^pg/mL.

### Factors associated with hypovolemic hyponatremia

Comorbidities and concomitant treatments associated with the presence of HH are presented in **Table 2**. HH cases had higher rates of treatment with at least one drug interfering with the RAAS than non-HH: 88.5% vs. 70.6% (p=0.017). In fact, in the entire cohort, we found a negative correlation between SNa levels and the total number of drugs in use interfering with either aldosterone secretion or mineralocorticoid action at diagnosis (r=-0.315, p=0.001). Furthermore, the higher the number of these drugs, the more likely the development of HH (OR: 1.4, 95%CI: 1.1 to 1.7). All 8 cases receiving simultaneous therapy with heparin and trimethoprim developed HH, representing 13.1% of HH cases, whereas no non-HH case received this treatment. Furthermore, the rates of both ResF and ALowF were higher in HH than in non-HH cases: 82% vs. 64.7% (p=0.038) and 85.2% vs. 68.6% (p=0.035), respectively. Likewise, the presence of CombF was also more frequent in HH cases: 70.5% vs. 45.1% (p=0.007).

A MLRA including the variables age ≥ 65 years, sex, over 10 years of evolution of DM, malnutrition, PDH, concomitant treatment with short-term glucocorticoid-therapy, any diuretic use, heparin, and MRB found that only malnutrition and MRB therapy were independently associated with HH (OR: 4.6, 95%CI: 1.8 to 11.6, and OR: 9.5, 95%CI: 1.1 to 79, respectively). Another model including age ≥ 65 years, sex, and the compound variables ResF, ALowF and CombF, found that only CombF remained independently associated with HH (OR: 2.9, 95%CI: 1.3 to 6.3).

### Factors associated with metabolic acidosis

MA at diagnosis was associated with a SK ≥ 6 mmol/L (p =0.017). In fact, all 8 cases who had the latter also had MA. Neither the presence of HH nor a SNa ≤ 125 mmol/L were associated with MA, nor an age ≥ 65 year nor sex. Of the comorbidities and pharmacological treatments shown in the **Table 2**, only CKD was statistically more frequent in MA cases than in non-MA: 57.1% vs. 30.4% (p=0.046). Of the studied compound variables regarding drugs or factors related with RAAS, the presence of ResF was the only one found at a higher rate in cases who developed MA than those did not: 91.4% vs. 69.6% (p=0.040). In fact, the higher the number of ResF, the lower the HCO3 values (r=-0.281, p=0.033).

### Causes and types of hypoaldosteronism

PAI was present in 7 /112 (6.3%) cases, 2 of which were due to congenital adrenal hyperplasia. The remaining 106 cases corresponded with acquired isolated hypoaldosteronism. Ninety (80.4%) of the 112 cases had at least one modifiable factor that could induce or trigger hypoaldosteronism. In fact, all cases with modifiable factors were receiving at least one drug interfering with the RAAS.

Aldosterone levels were measured in the presence of hyperkalemia in 51/112 (45.5%) cases. Hypoaldosteronism was found to be secondary to low aldosterone levels in 28/51 (54.9%), to mineralocorticoid resistance in 9/51 (17.6%), and to a combination of both in 14/51 (27.5%) cases. **Table 5** shows the comparison of clinical and biochemical characteristics when classifying by these 3 types of hypoaldosteronism. Cases with MRes tended to be hypovolemic and had non-significantly lower SNa values at diagnosis as well as a lower nadir SNa. Among the cases of AD, PAI was diagnosed only in 1/28 (3.6%). Excluding this case, the median cortisol levels for AD were 17 µg/dL [13-21.2], with ACTH levels 22.6 pg/mL [16.4-31.9].

**Table 5:** Clinical/Biochemical characteristics of the types of acquired hypoaldosteronism .

	Aldosterone deficit (N=28)	Mineralocorticoid resistance (N=9)	Combination of mineralocorticoid deficit/resistance (N=14)	*p*
Hypovolemic HNa, n (%)	10 (35.7)	7 (77.8)	8 (57.1)	*0.07*
Metabolic acidosis, n (%)	8/17 (47.1)	4/7 (57.1)	5/8 (62.5)	*0.747*
Short-term.GC, n (%)	5 (17.9)	2 (22.2)	3 (21.4)	*0.941*
Aldosterone, pg/mL	43 [32-67]	317 [256-610]	128 [97-155]	<0.001*
Cortisol, µg/dL	16.8 [12.7-21.1]	15.2 [12.6-16.3]	17.4 [13.4-18.9]	*0.115*
ACTH, pg/mL	23.1 [18.2-33.8]	30.3 [21.4-33.9]	20.3 [15.6-26.6]	*0.819*
Zenith serum K, mmol/L	5.9 ± 0.5	5.7 ± 0.5	5.8 ± 0.5	*0.489*
Serum K, mmol/L	5.4 ± 0.5	5.4 ± 0.3	5.3 ± 0.3	*0.580*
Serum Na, mmol/L	134 ± 7	130 ± 5	135 ± 4	*0.155*
Nadir serum Na, mmol/L	124 ± 6	122 ± 5	126 ± 4	*0.325*
HCO3, mmol/L	23.1 ± 3.8	23.3 ± 3.3	22.3 ± 3.6	*0.828*
Urine K, mmol/L	30 ± 13	28 ± 15	33 ± 14	*0.615*
Urine Na, mmol/L	87 ± 3	38 ± 15	68 ± 30	0.003*
Urine Na/K ratio	3.24 ± 1.62	1.5 ± 0.62	2.27 ± 1.17	0.009*
TTKG	3.6 ± 1.3	4.8 ± 1.5	4.5 ± 1.2	*0.099*

HNa, hyponatremia; GC, glucocorticotherapy; Na, sodium; K, potassium; HCO3, bicarbonate; HMA, hyperchloremic metabolic acidosis; Uosm, urine osmolality; TTKG, trans-tubukar potassium gradient.

*p<0.005.

## Discussion

Our results indicate that hypoaldosteronism is relatively common in adult endocrinological clinical practice, and is usually isolated, acquired, multifactorial, and potentially preventable. Its most frequent clinical manifestations are hyperkalemia, hypovolemic hyponatremia, and metabolic acidosis. And its presence seems to manifest mainly in the elderly, with a similar distribution between sexes. As expected, all cases in our cohort had urine parameters indicative of a low/inadequate potassium excretion regardless of whether hyperkalemia was present or not in spite of a glomerular filtration rate > 30 ml/min/1.73 m^2^. Thus, the patients showed a descent of the FKE (which is expected to be > 30 in hyperkalemia (21) and > 7 in (normokalemia) (22), as well as a low Urinary K+/creatinine ratio (which is expected to be > 150 in hyperkalemia (23)), a reduced TTKG (which is expected to be > 7, both in hyperkalemia and eukalemia, and even higher in hypovolemia (24)), and an elevated value of Urinary Na+/K+ ratio (which is expected to be < 1 in hyperkalemia (25–27)).In our cohort, hyperkalemia was the most consistent finding, documented in 106/112 (94.6%) cases at some time during the hypoaldosteronism episode. Thus, although the vast majority of patients developed hyperkalemia, 6 patients, over 5%, did not. Weidman et al. (28), Zipser et al. (29), and Davenport et al. (30), have previously described frank isolated hypoaldosteronism in the absence of hyperkalemia. In fact, although hyperkalemia is emblematic of hypoaldosteronism (5, 8, 15, 31), it is not a constant, not even in the setting of a primary adrenal crisis (32–36). Therefore, the absence of hyperkalemia should not rule out the suspicion of hypoaldosteronism.

Why some patients with hypoaldosteronism do not developing hyperkalemia is currently unknown. We found no salient clinical or biochemical features distinguishing patients who at no time had detected hyperkalemia from those who did, with a sole exception of a lack of factors related to mineralocorticoid resistance in 5/6 patients. One possible explanation for the absence of hyperkalemia might be that the action of other adrenal mineralocorticoids, primarily cortisol could be sufficient to maintain an acceptable K homeostasis (37). A second mechanism has been proposed by Gagnon and Halperin: minimal aldosterone release into the bloodstream could permit sufficient potassium excretion to prevent hyperkalemia, especially when an adequate delivery of sodium at the distal level of the nephron is assured, guaranteeing Na+/K+ exchange (38). We believe the opposite scenario might also hold true: any factor either reducing distal sodium delivery, albeit minimally, or directly related with distal tubular damage, could also induce higher SK levels. This latter hypothesis is supported by our findings. Hypovolemia, a potent stimulus for increased sodium reabsorption in the proximal tubule of the nephron, concomitantly reducing distal Na delivery, conditioned higher rates of marked hyperkalemia (SK ≥ 6 mmol/L) in the current study. In fact, patients with HH showed significantly lower urinary sodium levels (UNa) than those not presenting HH, although in both cases UNa was elevated. A possible contribution of distal tubular damage to hyperkalemia could explain the absence of mineralocorticoid resistance factors in 5/6 patients without hyperkalemia since tubular damage can induce resistance (2). When the distal tubule is preserved, Na+/K+ exchange is feasible if the distal delivery of sodium is sufficient, and therefore hyperkalemia less likely.

Hypovolemic hyponatremia (HH) was observed in 61/112 (54.5%) cases, and, in 6 of them, was the sole electrolytic manifestation of hypoaldosteronism. The vast majority of HH cases had isolated hypoaldosteronism, with primary adrenal failure present in only 6/61 cases (9.8%). Some authors have questioned whether HH can develop in patients with hypoaldosteronism absent an associated cortisol deficit such as in Addison’s Disease (5, 15, 31, 39–41). Our results clearly indicate that not only can HH develop in isolated hypoaldosteronism, moreover, it is frequent. Furthermore, our review of prior studies on patients with isolated hypoaldosteronism (13, 29, 42–53) indicates that the detection of HH is not limited to the current study, although the total number of subjects included in these investigations was smaller, with a finding of HH in 11 of 28 patients in the largest series (29).

The induction of HH in isolated hypoaldosteronism can be explained by renal salt wasting, and subsequent hypovolemia. The latter would stimulate arginine-vasopressin (AVP) release *via* baroreceptors, originating hyponatremia (54). Thus, hypovolemia may be induced by hypoaldosteronism even in absence of glucocorticoid deficiency as was already observed by Zipzer et al. in critically ill patients (29, 30). This explanation is supported by our findings. In the current study, when hypovolemia was present, hyponatremia was more likely to occur, and SNa levels were lower in hypovolemia than euvolemia. Therefore, hypovolemia, induced by renal salt loss, would be the principal factor in the development of HH in patients with isolated hypoaldosteronism.

We found that MA/4RTA occurred in the 60.3% of the cases, similar to the rate previously reported by DeFronzo (5). Different explanations for the development of MA in hypoaldosteronism have been proposed (1). However, factors associated with its presence have not been previously documented. Based on our results, it is probable that the development of MA depends on factors linked to alterations in the distal tubule of the nephron, since only the presence of ResF was associated with MA, and the higher number of ResF, the lower the HCO3 values. Thus, when distal tubular damage is present, or the action of mineralocorticoids is blocked, collecting duct intercalated cells would be unable to adequately excrete H+.

Ninety (80.4%) of the 112 cases presented modifiable factors that interfered with the RAAS. All were receiving at least one RAAS-interfering medication, with ACEI/ARBs the most frequent (50.9% of the entire cohort), followed by heparin (25%), β-blockers (17.9%), trimethoprim (12.5%) and MRBs (11.6%). A higher number of these drugs was associated with the development of HH, and with lower SNa. The combination of ALowF and ResF was accompanied by an increased risk for HH. In fact, all patients receiving the AlowF heparin together with the ResF trimethoprim developed HH. Malnutrition was also independently associated with HH. A study of Dekker et al. (55) and a research recently published by Gomez-Hoyos et al. (56) found that malnutrition, especially when severe, is a risk factor for hyponatremia in hemodialysis patients and intensive care patients respectively. Malnourished patients are predisposed to develop water shifts from the intracellular to extracellular compartments due to an increased catabolism of organic phosphates in tissues (57), which would facilitate hyponatremia. Although the mechanism is not clear in our cases, low salt and fluid intake secondary to inadequate nourishment could also be involved and precipitate or exacerbate a negative sodium balance. In any event, the importance of medication and malnutrition in the development of isolated hypoaldosteronism-induced HH indicates that this disorder is potentially preventable, and close patient monitoring called for when these risk factors are present.

Another finding of note is that most of the patients of our cohort were ≥ 65 years old, with a median age of 77 years, somewhat older than patients reported by DeFronzo et al (5). Healthy elderly people are known to have lower basal and stimulated aldosterone and renin levels than young people, and the response to hyperkalemia of the former is less effective than that of the latter (58). In fact, a progressive loss of CYP-11β2 (aldosterone synthase) function in the adrenal glomerulosa of the elderly has been described (59). Likewise, FKE decreases with age, reaching values even lower than in young CKD patients (60). This issue could facilitate the development of hypoaldosteronism-related hyperkalemia in the elderly. Michelis (61) explained that hyporeninemic hypoaldosteronism seemed to be more frequent in older patients due to an expansion of intravascular volume and consequent increase in atrial natriuretic peptides, both of which would inhibit renin secretion. In this setting, the use of drugs interfering with RAAS would more easily trigger hyperkalemia than in subjects with a normal renin level. A study of Murray et al. (62) demonstrated, in fact, that FKE decreased more noticeably in elderly than in young adults treated with NSAIDs. Likewise, a study of Gentry and Nguyen found that an age above 60 years is a risk factor for trimethoprim-related hyperkalemia, a type of hypoaldosteronism due to mineralocorticoid resistance (63). Just as the reduced values of aldosterone observed in elderly would facilitate the development of hyperkalemia on the one hand, so would they also facilitate the development of HH on the other hand. In fact, in 1987, Ishikawa (44) first described a type of HH in the elderly that improved with fludrocortisone, and was denominated “mineralocorticoid responsive hyponatremia of the elderly (MRHE).” A systemic review of all MRHE cases published through 2017 (45) found that the all 27 patients from the 9 eligible case reports referring to this condition had low blood aldosterone and renin levels, as well as elevated AVP and UNa levels, and a random blood cortisol above 13.2 ug/dL, with the exception of one case with a cortisolemia of 9 ug/dL. These findings suggest that MRHE could be actually a type of isolated hypoaldosteronism that occurs in elderly subjects in whom hypovolemia-induced renin and aldosterone production would be impaired, whereas AVP production remains unaffected. Furthermore, the incidence of hyponatremia is known to increase with age (64). Several mechanisms explain this fact: renal capacity to eliminate free water decreases, AVP production is augmented, total body water and intravascular volume are reduced (65), and polypharmacy and age-related diseases are more frequent. Polymedication could in fact be an important factor in an increased presentation of isolated hypoaldosteronism as a whole, and not just a risk factor for the development of hyponatremia. Therapies that can interfere with the RAAS include but are not limited to RAAS-interfering hypertension medication. In fact, with age comes an increase in hospital admittance, and thus the use of prophylactic heparin, which will often be continued following discharge, such as in the setting of orthopedic surgery. Last, but not least, the elderly, particularly when hypertensive, often eat low-salt diets. This could result in an insufficient delivery of sodium to the distal nephron, directly compromising the needed exchange of Na+ for K+, particularly when inadequate aldosterone stimulation or action interfere with the number or function of the amiloride-sensitive epithelial Na+ channels. All of the aforementioned factors could help explain an increased frequency of isolated hypoaldosteronism in the elderly.

Our findings also indicate the relevance of volemic status for the clinical manifestations of hypoaldosteronism, since those hypovolemic had a higher probability of showing a SK ≥ 6 mmol/L, SNa ≤ 125 mmol/L, and HCO3 ≤ 20 mmol/L, as well as the combination of hyperkalemia, HH and MA. As discussed above, maintenance of euvolemia would limit proximal tubular sodium reabsorption, permitting an adequate distal delivery of sodium, thus facilitating Na+/K+ exchange. Thus, both elevation of SK and SNa descent would be limited. Since hypovolemia would be treated with isotonic or hypertonic saline infusion, whereas euvolemia would not, a correct volemic classification in hypoaldosteronic patients would be essential for adequate therapy.

Our study has several weaknesses. The first is its retrospective design. Second is the absence of a current consensus on the diagnostic criteria of hypoaldosteronism in the scientific community. Third, the absence of a control group impedes the generation of major hypotheses regarding the causes and prevalence of hypoaldosteronism. The major strengths of the study reside in the detailed precision in case selection and case homogeneity, as well as a complete characterization of their clinical presentation and related factors. Furthermore, to our knowledge, the current study presents the largest series of hypoaldosteronism cases described in a single study since the entity was first identified in 1957.

In conclusion, we can confirm that isolated hypoaldosteronism is a clinical disorder characterized by a diminished urinary potassium excretion with varying degrees of renal sodium loss, which predispose not only to the development of hyperkalemia and MA, but also to that of HH. We therefore believe acquired isolated hypoaldosteronism should always be included in the diagnostic algorithm of hypovolemic hyponatremia. Finally, we feel that patients receiving RAAS-interfering medication, as well as patients with malnutrition, should be closely monitored, to permit early detection of the development of hypoaldosteronism.

## Data availability statement

The data supporting the conclusions of this article will be made available by the authors upon reasonable request.

## Ethics statement

The study complied with accepted standards of good clinical practice according to the Helsinki Declaration and was approved by the Ethical Committee of the HCSC (Cod. 20/714-E_BS, December 14^th^-2020). Written informed consent was waived.

## Author contributions

Conceptualization: JGR-S, IR; Methodology: JGR-S, ALC-P, MPDMN, MAR-H, EG-H; Validation: JGR-S, IR; Formal analysis: JGR-S; Investigation: JGR-S; Writing – Original Draft Preparation: JGR-S, IR; Writing – Review & Editing: JGR-S, ALC-P, MPDMN, MAR-H, EG-H, IR; Supervision and reviewing: IR, ALC-P, MPDMN, MAR-H; Resources: JGR-S, ALC-P, IR. 

## Funding

This research received a grant of the Sociedad de Endocrinología, Nutrición y Diabetes de la Comunidad de Madrid (SENDIMAD) through the “Beca de Ayuda a la Investigación SENDIMAD 2020” awarded on November 25, 2020, in Madrid, Spain. During the period of data collection, JR-S was hired as a clinical investigator by the Foundation for Biomedical Research at the Hospital Clínico San Carlos (Reference number. INV-15-2019).

## Acknowledgments

We thank the Fundación Para la Investigación Biomedica de El Hospital Clínico San Carlos of Madrid, for the logistical support in the publication process.

## Conflict of interest

The authors declare that the research was conducted in the absence of any commercial or financial relationships that could be construed as a potential conflict of interest.

## Publisher’s note

All claims expressed in this article are solely those of the authors and do not necessarily represent those of their affiliated organizations, or those of the publisher, the editors and the reviewers. Any product that may be evaluated in this article, or claim that may be made by its manufacturer, is not guaranteed or endorsed by the publisher.
