# Supplementary material for: Clinical manifestations and associated factors in acquired hypoaldosteronism in endocrinological practice
Source: Front Endocrinol (Lausanne). 2022 Oct 11;13:990148. doi: 10.3389/fendo.2022.990148 (PMC9592828; doi:10.3389/fendo.2022.990148)
Supplement: Supplementary file 1 [file DataSheet_1.docx]

Supplementary Material

# Supplementary Data

1. **COLLECTED CLINICAL AND BIOCHEMICAL VARIABLES**

Volemic status, levels of serum cortisol, plasma ACTH, serum aldosterone, plasma renin concentration, serum sodium, serum potassium, serum chlorine, serum creatinine, serum urea, glycemia, plasma osmolality, venous/arterial whole blood pH, pCO2, blood bicarbonate, urine creatinine, urine sodium, urine potassium, urine osmolality, calculated trans-tubular potassium gradient, Urine potassium-to-creatinine ratio, fractional excretion of potassium, serum-urine potassium ratio, and urine sodium-to-potassium ratio.

1. **CREATED COMPOUND VARIABLES**

**2.1 Comorbidities and concomitant treatment variables categorized as non-modifiable or modifiable factors:**

****Non-modifiable factors:***

hypertension, diabetes mellitus, chronic kidney disease, obstructive uropathy, renal transplant, alcoholism, and a prior event of dilutional hyponatremia.

****Modifiable factors:***

Urinary tract infection, malnutrition, self-reported low-sodium diet, primary adrenal insufficiency, short-term and chronic glucocorticoid therapy, use of heparin, trimethoprim, cyclosporine, tacrolimus, non-steroidal anti-inflammatory drugs, β-blockers, aliskiren, angiotensin-converting enzyme inhibitors or angiotensin 2 receptor blockers, mineralocorticoid receptor blockers, loop diuretics, thiazide, and thiazide-amiloride diuretics.

**2.2 Comorbidities and concomitant treatment variables categorized according to their interaction with mineralocorticoid homeostasis.**

****Aldosterone-lowering factors* (ALowF)*:***

Diabetes mellitus, chronic kidney disease, renal transplant, chronic glucocorticoid-therapy, use of heparin, non-steroidal anti-inflammatory, β-blockers, aliskiren, and angiotensin-converting enzyme inhibitors or angiotensin 2 receptor blockers.

****Mineralocorticoid-resistance factors* (ResF)*:***

Renal transplant, obstructive uropathy, urinary tract infection, use of trimethoprim, cyclosporine, tacrolimus, and mineralocorticoid receptor blockers

****Any combination of both described above* (CombF)*:***

When at least one factor from each group was present

**2.3 Drugs grouped** **as interfering with the renin-angiotensin-aldosterone system (RAAS).**

Trimethoprim, cyclosporine, tacrolimus, heparin, non-steroidal anti-inflammatory, β-blockers, chronic glucocorticoid-therapy, aliskiren, angiotensin-converting enzyme inhibitors, angiotensin 2 receptor blockers, mineralocorticoid receptor blockers, amiloride diuretic.
